# Are Buccal Fat Pad–Derived Stem Cells Effective in Adults for Maxillary Bone Regeneration? A Systematic Review

**DOI:** 10.1097/SCS.0000000000012186

**Published:** 2025-11-24

**Authors:** Pierpaolo De Francesco, Paolo Vescovi, Ilaria Giovannacci

**Affiliations:** Department of Medicine and Surgery,Oral Medicine and Oral Surgery Laser Unit, University Centre of Dentistry, School of Specialty in Oral Surgery, University of Parma, Parma, Italy

**Keywords:** Adipose tissue, bone regeneration, guided tissue regeneration, oral surgery, stem cells

## Abstract

Critical-size bone defects in the maxillofacial region remain a major challenge. Although autografts are widely used, they are limited by donor-site morbidity and graft availability. Tissue engineering with mesenchymal stem cells, particularly buccal fat pad-derived stem cells (BFPSCs), offers a promising alternative due to their accessibility and osteogenic potential. This systematic review assesses clinical evidence on the use of BFPSCs for regenerating oro-maxillofacial bone defects in adults. A systematic search was conducted in PubMed/MEDLINE, EMBASE, and Cochrane databases for studies from January 2015 to September 2025. Inclusion criteria were in vivo human studies using BFPSCs for maxillofacial bone regeneration. Excluded were in vitro, animal studies, cell-free BFP applications, and non-original articles. Two reviewers independently screened studies; discrepancies were resolved by a senior reviewer. The level of evidence was assessed using Oxford CEBM criteria. Out of 375 identified articles, 6 clinical studies (including one randomized trial) involving 49 patients met inclusion criteria. BFPSCs were applied alone or with autologous bone. All studies reported favorable clinical and radiologic outcomes; some included histologic data confirming new bone formation. BFPSCs demonstrate promising potential for maxillofacial bone regeneration. However, high-quality studies are needed to confirm effectiveness and define standardized protocols.

Regenerative medicine, focused on restoring tissues and organs, finds in mesenchymal stem cells (MSCs) a powerful ally for achieving clinically significant results.^1–3^ MSCs are multipotent adult stromal stem cells found in various tissues (eg, bone marrow, periosteum, vessel walls, adipose, muscle, tendon, peripheral circulation, umbilical cord blood, skin, and dental tissues). They possess the capacity for self-renewal and differentiation into different cell lineages under specific conditions, contributing to tissue repair. Furthermore, they present homing properties and paracrine actions.^4,5^ For these reasons, stem cells are frequently used in conjunction with biomaterials/scaffolds and growth factors to expedite bone healing, enabling more rapid and effective bone regeneration.^6–8^

A critical-sized bone defect is defined as a bone defect that is too large to heal spontaneously or with a standard cancellous bone graft.^9^ From a clinical point of view, these defects represent the smallest bone losses that will not heal during the spontaneously. However, the reconstruction and repair of bone tissue in the mouth, face and jaws in implant surgery it remains a challenging situation for most oral and maxillofacial surgeons.^10^ In particular, the fields where it remains most challenging are post-extraction bone reconstruction,^11^ treatment of jawbone diseases (such as trauma, infection, and neoplastic diseases),^12^ and orthognathic surgery.^13^

Bone marrow-derived mesenchymal stem cells (BMSCs) have been utilized as a cell source within the field of bone tissue engineering to address bone defects in the maxillofacial region.^14–16^ However, clinical use of BMSCs is hindered by several limitations; their harvest can be invasive and uncomfortable.^17^

These limitations have been partly overcome using adipose-derived stem cells (ADSCs). Adipose tissue, which is abundant and easily accessible, provides significantly more stem cells than bone marrow, ~500 times than an equivalent volume of bone marrow.^18^ Furthermore, ASCs show less senescence and greater proliferative capacity than BMSCs.^19^ However, harvesting adipose tissue from sites such as the gluteus, thighs, and abdomen also has its drawbacks, such as heterogeneity of harvested cell populations, insufficient aspirate volume, donor-site morbidity, painful surgery, and postoperative outpatient complications.^20^

A novel source of Adipose-Derived Stem Cells has emerged in the maxillofacial region: buccal fat pad–derived stem cells (BFPSCs), a novel subset of ADSCs. These stem cells have shown significant potential for the reconstruction of critical maxillofacial bone defects.^20^

Unlike conventional harvesting procedures, the buccal fat pad represents an intraoral and minimally invasive source of ADSCs, with reduced morbidity, shorter operative times, and no visible scarring, which is particularly advantageous in maxillofacial applications.

The buccal fat pad (BFP), or Bichat’s fat, is a significant collection of fat within the midface, it is encapsulated, and it differs from subcutaneous fat. It plays various roles, including aiding in sucking, supporting chewing, protecting nerves and blood vessels, separating chewing muscles, and contributing to facial aesthetics.^21^ The BFP offers a straightforward and dependable solution for reconstructing various intraoral defects. Its rich blood supply and proximity to these defects make it particularly advantageous. The surgical technique is relatively simple and boasts a high success rate in diverse applications, it can be used both as a pedicular or free graft, such as closing oroantral fistulas, correcting congenital defects, treating jawbone necrosis, and reconstructing defects resulting from tumor removal.^22,23^

Despite the growing interest in BFPSCs, there is a gap of knowledge of literature exploring their clinical outcomes. Previous reviews have mainly emphasized biological potential or in vitro findings, with limited attention on clinical application.

The aim of this systematic review is to investigate effectiveness of BFPCs in maxillary bone regeneration. Evidence that BFPCs are effective both from a histologic and a clinical point of view would lead to their consideration as a therapeutic option in maxillary bone regeneration.

## MATERIALS AND METHODS

### Protocol Development and Eligibility Criteria

This systematic review was organized and reported according to the recommendations of the PRISMA (reporting items for systematic reviews and meta- analysis) Extension Statement for Reporting of Systematic Reviews Incorporating Network Meta-analyses of Health Care Interventions.

All in vivo studies that utilized BFPSCs and BFP-DFAT cells from human sources were included to investigate bone regeneration of maxillofacial bone defect. The principal outcome of the review was to evaluate effectiveness of BFPCs by examining these treatment endpoints: clinical assessment, histologic evaluation and/or radiologic analysis.

The following focused question was formulated: are buccal fat pad–derived stem cells effective in adults for oro-maxillofacial bone defect regeneration?

Inclusion criteria for articles were based on the following PICOS:

P (Problem / population): All in vivo studies that utilized BFPSCs and BFP-DFAT cells to investigate bone regeneration of oro-maxillofacial bone defect.

I (Intervention): Treatment of maxillofacial bone defect with buccal fat pad–derived stem cells (BFPSCs).

C (Comparison): Use of BFPSCs was compared with any type of treatment or no treatment.

O (Outcome): Effectiveness.

S (Study design): Any type of study design.

### Information Sources and Search

Relevant studies published between January 2015 and September 2025 were identified through searches of the PubMed/MEDLINE, EMBASE, and Cochrane electronic databases.

For PubMed/MEDLINE, the following search string was used: A combined search strategy utilizing Medical Subject Headings (MeSH terms) and free-text keywords:

(([“Stem Cells”[Mesh] OR “Mesenchymal Stem Cells”[Mesh] OR Stem Cell* OR “Mesenchymal Stem Cell*“] AND [“buccal fat pad” OR “BFP” OR “BFPSCs” OR “Bichat’s fat pad” OR “Buccal Fat Pad-Derived Stem Cells” OR “adipose stem cells”]) AND ([“Bone Regeneration”[Mesh] OR “Guided Tissue Regeneration, Periodontal”[Mesh] OR “Guided Tissue Regeneration”[Mesh] OR “Tissue Engineering”[Mesh] OR “Bone Regeneration” OR “Guided Tissue Regeneration, Periodontal”[tiab] OR “Guided Tissue Regeneration” OR “Tissue Engineering”])).

For EMBASE and Cochrane Library, equivalent free-text keywords were used and adapted to each database, covering the same core.

During the database searches no automatic filters were applied for humans or language. Instead, restrictions to human studies and English language were applied during the manual screening stage, as specified in the inclusion criteria.

### Study Selection and Data Collection

To determine the inclusion of articles in the review, a double evaluation was carried out: first, the titles and abstracts were examined by 2 independent reviewers (P.D.F. and I.G.); then, articles deemed potentially relevant were subjected to a thorough full-text analysis.

Given the importance of not leaving out any potential evidence, it was decided to include abstracts with poorly defined results in the full-text analysis, while being aware of the greater interpretative complexities involved.

Inclusion criteria for the title and abstract analysis were the following:

- Manuscripts published in English between January 2015 and September 2025.
- Articles with full text.
- Patients with oro-maxilofacial bone defects treated with buccal fat pad–derived stem cells (BFPSCs) alone or in combination with other biomaterials.
- In vivo studies BFPSCs from human sources.
  Exclusion criteria for title and abstract analysis were:
- Not original study (abstract, guidelines, and letters).
- Original studies were excluded if they involved BFP flaps or masses (cell-free).
- In vitro studies.
- Non-human source.
  Full-text articles of all potentially relevant studies were retrieved and independently assessed by 2 reviewers (P.D.F. and I.G.) based on inclusion criteria. Discrepancies were resolved through discussion with a senior reviewer (PV). Inclusion of articles in the review required adherence to all inclusion criteria and assessment of at least one of these outcomes: clinical, radiologic, and/or histologic. The same inclusion criteria were applied to the full-text analysis, together with absence of reporting of any of the studied outcomes.
  Primary outcomes included:
- Clinical outcomes: evaluation of healing, absence of pain, infection, inflammation, or wound dehiscence.
- Radiologic outcomes: bone formation and graft integration assessed by imaging techniques such as cone beam computed tomography (CBCT) or orthopantomograms.
- Histologic outcomes: microscopic analysis of bone tissue regeneration through biopsy samples.

Secondary outcomes involved assessment of adverse effects, patient morbidity, hospital stay duration, and patient discomfort.

### Level of Evidence

The analysis included 6 in vivo studies on humans, all of which were assessable according to the Oxford CEBM guidelines.^20,24–28^

The level of evidence was 2b for 1 study,^25^ 3b for 3 studies,^24,26,28^ and 4 for 2 studies.^20,27^

### PRISMA Flow Diagram

**FIGURE 1:**
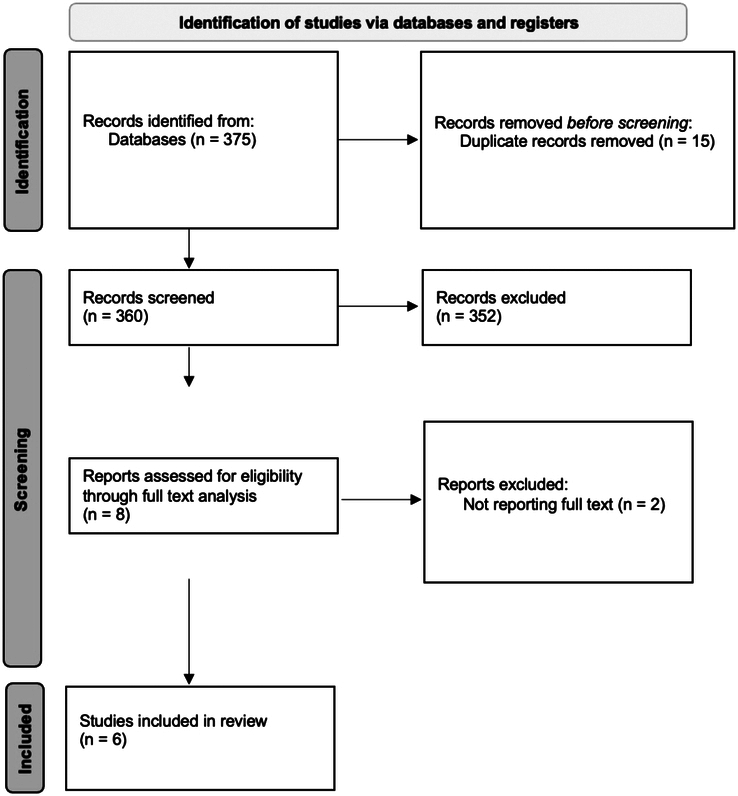
Search strategy flowchart.

The study selection process is detailed in the PRISMA 2020 flow diagram, now presented as Figure 1.

### Risk of Bias Assessment

To assess the methodological quality and risk of bias of the included diagnostic accuracy studies, we used the ROBINS-I (risk of bias in non-randomized studies-of interventions).

## RESULTS

### Study Selection

A total of 375 citations of articles published in English between January 2015 and September 2025 were identified for inclusion in the review. After removing duplicates, 360 articles remained for analysis. 352 articles were excluded after the screening of titles and abstracts. Full-text analysis was conducted on the remaining 8 articles, and 2 were further excluded as it was not possible to obtain the full article. Six articles were included in the final review.^20,24–28^

### Risk of Bias Assessment

**FIGURE 2:**
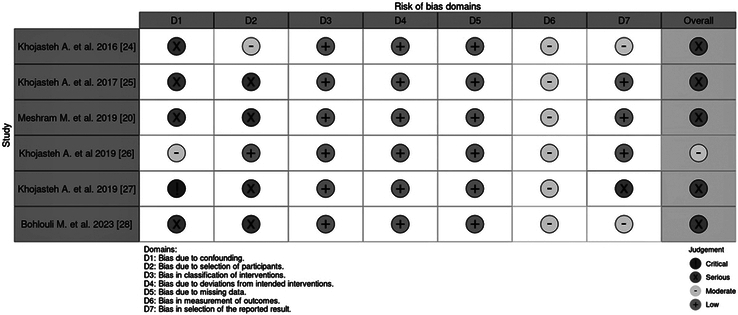
ROBINS-I (risk of bias in non-randomized studies-of interventions).

The risk of bias for included studies has been performed using the ROBINS-I tool, with results summarized in Figure 2. Among the 6 included studies, 5 were judged to be at serious risk of bias, and 1 was assessed as having a moderate risk of bias. The predominance of serious risk of bias suggests that the results of this systematic review should be interpreted with caution.

### Population and Studies Characteristics

The study population consisted of 49 patients, 29 males and 20 females. The mean age of the participants was 42.8 years (18–65 y) (Supplemental Table 1, Supplemental Digital Content 1, http://links.lww.com/SCS/I754). In all selected studies,^20,24–28^ patients were excluded if they had systemic medical conditions that contraindicated surgical intervention, or conditions known to impair wound healing (eg, smoking). Thirty out 49 patients (61.2%) patients were treated with the use of BFPSCs, whereas 19 out of 49 (38.8%) were treated with traditional bone regeneration techniques such as autologous bone grafts or xenografts, with membranes and particulate materials, as described in the control groups of the included studies.

Out of the 49 patients, 30 (61.2%) were treated with buccal fat pad–derived stem cells (BFPSCs), whereas 19 (38.8%) received traditional bone regeneration methods, including autologous or xenogenic grafts combined with membranes and particulated materials. BFPSCs were used alone in 2 studies;^20,28^ in others, BFPSCs were combined with autologous bone harvested either from the lateral ramus cortical plate (LRCP) or the anterior iliac crest (AIC). Control groups across studies received autologous bone grafts without stem cells, harvested from LRCP or AIC.

### Evaluation of Treatment Outcome

Assessment of treatment success (Supplemental Table 2-3, Supplemental Digital Content 1, http://links.lww.com/SCS/I754) of BFPSCs use was carried out through the evaluation of clinical, radiologic, and/or histologic outcomes.

All included studies reported clinical and radiologic outcomes,^20,24–28^ whereas 4 out of the 6 included studies also present histologic outcomes.^20,24,25,27^

Regarding clinical endpoints, the variables analyzed were soft tissue healing, graft integration, and the absence of adverse events (eg, pain, inflammation, infection, dehiscence). The mean follow-up after surgery, was 15 months (minimum 5–maximum 48). The goal was to assess healing of soft and grafted tissues, looking for the absence of unexpected pain, local inflammation, infection, and wound dehiscence. BFPSC-treated sites consistently demonstrated satisfactory healing.

Regarding radiologic outcomes, all studies used cone beam computed tomography (CBCT) and/or orthopantomograms for postsurgical radiologic evaluation. Five studies^24–28^ performed CBCT scans before surgery at 5, 6, and 12 months after baseline. One study^20^ performed CBCT scans at 3, 6, and 12 months after surgery. The radiographic assessment revealed integration of the grafted tissue and proper 3-dimensional bone formation.

Regarding histologic outcomes, 4 studies performed histologic and / or cytologic analysis.^20,24,25,27^ In addition,^20^ performed a flow cytometry to analyze cell surface markers on adipose-derived stem cells (ASCs) twice: once on cultured ASCs and again at 3 months post-surgery on bone biopsy tissue. The results showed consistent expression of HLA-ABC, CD29, CD49e, CD51, and CD90, with variable expression of CD49d, CD9, CD34, CD105, and CD166. ASCs were negative for MHC-II (HLA-DR), CD40, CD40L, CD80, and CD86, and inhibited lymphocyte proliferation. These negative markers remained consistent after differentiation. CD54, CD49, and CD34 were exclusively expressed, indicating successful ASC isolation.

Regarding secondary endpoints, 2 studies^26,28^ compared BFPSC-based harvesting with autologous bone harvesting (LRCP and AIC) in terms of patient-reported outcomes. BFPSC-treated patients experienced less postoperative pain, shorter hospital stays, and lower morbidity. These findings suggest a potential advantage of BFPSCs in terms of patient comfort.

## DISCUSSION

This review explores the applications of buccal fat pad stem cells (BFPSCs) in bone engineering, focusing on clinical, radiologic, and histologic outcomes in vivo.

Regenerative medicine uses a variety of approaches—including allogeneic or autologous cells, blood components, biomaterials, gene modifications, and tissue engineering techniques—individually or in combination, to stimulate tissue repair.^29–31^

Tissue engineering offers a promising approach to tissue repair by using a patient’s own cells, in conjunction with scaffolds and stimulating signals, to regenerate damaged tissue. This personalized approach avoids the complications of immune rejection and the need for anti-rejection medications.^32^

Oral and maxillofacial surgery often involves reconstructing critical-sized osseous defects—defects too large for self-healing, which remains a challenge to this date.^33^ Etiologies include trauma, degenerative diseases, jawbone tumors, and infections. Beyond structural damage, these defects cause bone dysfunction, deformities, and significantly impact patient quality of life.^34^

Stem cell tissue engineering holds considerable promise for the repair of extensive jawbone defects. This potential stems from the advantageous characteristics of stem cells, including their ease of isolation, remarkable flexibility, immunomodulatory properties, and multipotent differentiation capacity.^35–37^ Stem cell candidates include embryonic stem cells, induced pluripotent stem cells (reprogrammed somatic cells), and adult stem cells. Adult stem cells, such as mesenchymal stem cells (originally found in bone marrow), can be sourced from various tissues, including fat, skin, bone marrow, blood, and skeletal muscle.^38^

MSCs are widely used in cranio-maxillofacial tissue engineering due to their secretome and differentiation capacity.^39^ As medicine increasingly embraces personalized and cell-based approaches, MSCs, with their therapeutic potential are poised to become a cornerstone of regenerative therapies.^40–42^ Although MSCs are commonly harvested from bone marrow (specifically the femur, tibia, and pelvic bone), this procedure presents several drawbacks: technical complexity, prolonged surgical times, donor-site morbidity requiring a second surgical site, and extended postsurgical pain.^43^ Fortunately, MSCs can also be isolated from various orofacial tissues, such as dental pulp, apical papilla, periodontal ligament, exfoliated deciduous teeth, and buccal fat pad; researchers have compared MSCs from oral tissues to MSCs from non-orofacial bone marrow and found that they have comparable multipotent differentiation capacity, as shown by MSC-specific marker expression and in vivo bone formation.^44–48^

Adipose-derived stem cells (ADSCs) offer an alternative source due to ease of harvesting, high cell yield, and low donor-site morbidity.^38^ Subcutaneous adipose tissue, especially that of the abdomen, thigh, and arm, is the most clinically relevant source of ADSCs, as it produces large numbers of cells. The multilineage capacity of ASCs makes them a valuable tool for both cell therapy and tissue engineering.^19^

ADSCs secrete a wide array of paracrine factors, including inflammatory cytokines, angiogenic, trophic, and growth factors, which contribute to tissue repair, wound healing, and organ regeneration. ADSCs release membrane-derived vesicles (MVs) containing growth factors, cytokines, RNAs, and miRNAs, enabling them to communicate with distant cells. These MVs, carrying osteogenic factors like BMP-2, contribute to bone regeneration through paracrine effects.^49^ ADSCs can differentiate into various lineages, including adipocytes, chondrocytes, osteoblasts, cardiomyocytes, skeletal muscle cells, neurons, hepatocytes, and tenocytes; in vitro ADSCs differentiation is induced using selective media containing lineage-specific induction factors.^50,51^ In addition, recent research has focused on designing novel 3D biomaterials that combine ADSCs with biomimetic scaffolds [such as β-tricalcium phosphate (β-TCP), bioactive glass (BAG), platelet-rich plasma (PRP)], made from either natural or synthetic materials. These innovative 3D constructs have shown considerable promise for promoting tissue repair and organ regeneration.^52,53^

ADSCs are differentiated into osteoblasts over 14 days in a medium containing DMEM (10% FBS), β-glycerol phosphate, gentamicin, dexamethasone, L-glutamine, dihydroxyvitamin D3, and ascorbic acid. The medium is replaced every 3 days. After fixation, calcium phosphate in the ECM is assessed with Alizarin Red or von Kossa. Osteogenic differentiation is confirmed by upregulation of Runx-1, BMP-2, BMP-4, type I collagen, osteoponin, alkaline phosphatase, osteocalcin, bone sialoprotein, and parathyroid hormone receptor.^38^ ADSCs which possess significant osteogenic potential, osteoinductive capacity and immunomodulatory properties, have found clinical application in the difficult field of bone regeneration, a complex process susceptible to numerous influencing factors.^54–56^ Lendeckel et al^57^ first used ADSCs clinically in 2004, reconstructing widespread calvarial defects in a 7-year-old girl after a severe head injury, good new bone formation was achieved after 3 months. Sándor GK et al^58^ used a tissue-engineered of β-TCP granules, BMP-2, and autologous ADSCs, to reconstruct a mandibular defect, restoring the chin’s original anatomy.

After the initial clinical application of ADSCs, numerous studies in bone tissue engineering have provided clear evidence of their crucial role in the treatment of cranio-maxillofacial defects. This effectiveness is attributed to their high ability to differentiate towards the osteogenic lineage.^59–61^ Although ADSCs show promise for treating severe bone defects, donor age negatively impacts their osteogenic potential, limiting their therapeutic efficacy. ADSCs from younger donors exhibit higher expression of osteogenic markers (OPN, OCL, BMP-2) and greater calcium deposition compared with those from elderly patients. Therefore, donor age should be considered when using ASCs for cranio-maxillofacial bone reconstruction.^59^ Moreover, even harvesting has drawbacks, including cell heterogeneity, insufficient aspirate volume, donor-site morbidity, and postoperative complications.^20^

BFPSCs have emerged as a promising source of adipose-derived stem cells. Harvesting is simple, minimally invasive, and associated with low donor-site morbidity.^62^ These cells have been characterized by a high rate of osteogenic differentiation and their use in the management of critical defects of the jaw bones has been successful.^63^

The buccal fat pad (BFP), a distinct anatomical structure in the cheek, was first documented by Heister ~300 years ago; it is a specialized adipose tissue, and its rich vascularization makes it highly versatile for various applications in reconstructive oral surgery.^64^ Furthermore, the buccal fat pad represents an easily accessible source with minimal post-harvest morbidity, has a constant size that is not influenced by variations in fat distribution, body weight, or age, and contains abundant neural-crest-derived stem cells with a faster proliferation rate and higher expression of osteogenic and angiogenic surface markers, compared with stem cells isolated from other adipose sources.^28,65^ BFPSCs, originating from cranial neural crest cells, may contribute to the promising regenerative outcomes observed in maxillary bone reconstruction and support further exploration of their translational application.^66^

Analysis of selected in vivo studies revealed encouraging results for both the isolated use of BFPSCs and their combination with scaffolds or autologous bone grafts. Clinical data showed improved healing of bone defects, with reduced postoperative pain. Radiologic evaluations confirmed the formation of new bone tissue, with adequate integration with the surrounding bone. Histologic examinations revealed the presence of well-structured newly formed bone tissue with good vascularization and normal cellularity.

To confirm these preliminary results, it is necessary to design standardized trials that reduce the biases currently present in studies investigating this technique.

BFPSCs may represent an adjunctive treatment for bone regeneration in maxillary defects due to their ease of harvesting and ability to promote bone regeneration. Therefore, donor variability represents a critical factor influencing stem cell quality and regenerative potential. Thus, further randomized controlled clinical trials with long-term follow-up are needed to confirm these preliminary results and optimize clinical application.

## Supplementary Material
