# Supplementary material for: Are Buccal Fat Pad–Derived Stem Cells Effective in Adults for Maxillary Bone Regeneration? A Systematic Review
Source: J Craniofac Surg. 2025 Nov 24;37(6):1455–60. doi: 10.1097/SCS.0000000000012186 (PMC13200894; doi:10.1097/SCS.0000000000012186)
Supplement: Supplementary file 1 [file scs-37-1455-s001.docx]

**Supplemental Tables**

Supplemental Table 1: Sample characteristics (n = 49 subjects)

| Variables | Outcome |
| --- | --- |
| Gender [*n* (%)]   - Female - Male | 29 (59.2%)  20 (40.8%) |
| Age (years)   - Mean - Standard deviation - Median - Range | 42.8  14.95  45  18 - 65 |
| Treatment location [*n* (%)]   - Mandible - Maxilla - Both | 28 (57.1%)  18 (36.7%)  3 (6.1%) |
| Use of BFPSCs [*n* (%)]   - Yes - No | 30 (61.2%)  19 (38.8%) |
| Underlying medical conditions [*n* (%)]   - Yes - No | 0  100 (100%) |

Supplemental Table 2: Results of the included studies.
BFPSCs: buccal fat pad derived stem cells; AIC: anterior iliac crest; CM: collagen membrane; LRCP: lateral ramus cortical plate; ABBM: anorganic bovine bone mineral; NBBM: natural bovine bone mineral, ABG: Autologous bone grafting; GBR: guided bone regeneration; BW1: Bone width values preoperative

| Authors | Study design | Number of patients | Treatment type | Bone defect size | Follow-up | Results |
| --- | --- | --- | --- | --- | --- | --- |
| Khojasteh A. et al. 2016 [24] | Prospective clinical study (preliminary study) | Test Group (BFPSCs + AIC): 4  Control Group (AIC): 4 | BFPSCs with autogenous iliac bone graft in treatment of maxillomandibular extreme jaw atrophy | BW1:  1.96 ± 0.98 mm  2.88 ± 1.12 mm | 5 months | Mean bone width change at the graft site: greater in the test group than in the control group (3.94–1.62 mm versus 3.01–0.89 mm).  New bone formation: 65.32% in the test group versus 49.21% in the control group.  Increased amount of new bone formation and decreased secondary bone resorption in extensively atrophic jaws. |
| Khojasteh A. et al. 2017 [25] | Prospective randomized clinical trial | First Group (AIC): 3  Second Group (LRCP + BFPSCs): 3  Third Group (AIC+BFPSCs): 4 | Lateral ramus cortical plate with BFPSCs in the Treatment of Human Alveolar Cleft Defects | 6.80 ± 2.41 mm^3^  7.99 ± 2.50 mm^3^  7.52 ± 2.91 mm^3^ | 6 months | LRCP+BFPSCs group demonstrated defect closure and greater amounts of new bone formation than AIC + CM group (75 ± 3.5% vs 70 ± 10.40%).  AIC+BFPSCs group showed the greater amounts of new bone regeneration (82.5 ± 6.45%).  Histological analysis of the LRCP+BFPSCs and AIC+BFPSCs groups revealed the presence of new lamellar bone with an osteoblastic rim. |
| Meshram M. et al. 2019 [20] | Prospective clinical study  (pilot study) | Test Group: 5  (BFPSCs) | Maxillofacial bone defects secondary to enucleation of jaw pathologies reconstructed by BFPSCs | 35 X 45 mm  25 X 45 mm  45 X 55 mm  25 X 35 mm  30 X 20 mm | 12 months | Histological and radiological analysis showed high bone density and architecture.  Clinically, excellent functional and aesthetic results were achieved, resulting in improved quality of life. |
| Khojasteh A. et al 2019 [26] | Prospective clinical study  (exploratory study) | Test Group (BFPSCs + ABBM): 7  Control Group (LCRP + ABBM): 7 | BFPSCs in combination with ABBM for vertical and horizontal augmentation of atrophic posterior mandibles | Not specified | 12 months | No statistically significant differences (P > 0.05) in bone volume formation (vertical and horizontal augmentation) between BFPSCs and autogenous particulate bone in combination with ABBM. |
| Khojasteh A. et al. 2019 [27] | Case series | 2 (BFPSCs **+** NBBM) | Reconstruction of large alveolar defects after extraction of multiple impacted teeth using BFPSCs in combination with NBBM | Large bone defect > 6 cm | 10 months  48 months | Proper healing and survival of dental implants was observed radiographically and clinically.  The application of BFPSCs can be considered an efficient treatment for bone regeneration in large alveolar bone defects.  Histological analysis showed the deposition of mineralized nodule-like structures and confirmed the osteogenic capacity of the cultured cells. |
| Bohlouli M. et al. 2023 [28] | Prospective clinical study  (pilot study) | Test Group (BFPSCs): 5  Control Group (ABG): 5 | Maxillofacial bone defects secondary to enucleation of jaw pathologies reconstructed by BFPSCs | Not specified | 12 months | Duration of hospitalization and pain in the ABG group were longer than test group.  Greater discomfort at the donor site in the control group.  Radiological evaluation showed favorable bone healing after one year in all patients.  Clinical evaluation has suggested that BFPCs is safe for patients with maxillofacial bone defects. |

Supplemental Table 3: Treatment Outcomes
NA: not applicable

|  | Treatment Outcomes | | |
| --- | --- | --- | --- |
| Authors | Clinical | Radiological | Histological |
| Khojasteh A. *et al.* 2016 | The surgical site healed well, with soft tissue and grafted tissue healing progressing normally over the bi-weekly evaluations | Five months after the surgical procedure a CBCT was obtained, showed bone width change at the graft site: greater in the test group than in the control group (3.94–1.62 mm versus 3.01–0.89 mm) | The histomorphometric analysis revealed that the scaffolds were incorporated into the new bone and had an osteoid matrix. |
| Khojasteh A. *et al.* 2017 | The surgical site healed well, with soft tissue and grafted tissue healing progressing normally over the bi-weekly evaluations | Six months post-procedure, CBCT confirmed successful new bone formation. | The histological analysis revealed the new lamellar bone with osteoblastic rim without inflammatory cells infiltration |
| Meshram M. *et al.* 2019 | Postoperatively, patients were evaluated for pain, edema, infection, altered sensation, and complications. Good healing | All the measurements for osseous regeneration like bone density, bone height was taken on OPGs by a single operator. Favorable bone regeneration | Postoperative histopathologic evaluation of all the patients was done by taking a bone biopsy from the operative site at 3 and 6 months. Favorable bone regeneration. |
| Khojasteh A. *et al.* 2019 | Post-operatively, the surgical site exhibited good healing. Healing was monitored weekly for the first month, and then monthly for the subsequent six months. | Six months post-procedure, CBCT confirmed successful new bone formation. | NA |
| Khojasteh A. *et al.* 2019 | Post-operatively, the surgical site exhibited good healing. Healing was monitored biweekly for the first month postoperatively and then monthly for 6 months. | Six months post-procedure, CBCT confirmed acceptable integration of bone substitute to the recipient healthy bone and 3-dimensional bone formation | Postoperative histopathologic evaluation confirmed the osteogenic capability of the cultured cells. Stem cells demonstrated a similar spindle-shaped morphology and attachment to the NBBM. |
| Bohlouli M. *et al.* 2023 | Evaluation demonstrated good healing without any unexpected pain, signs of local inflammation, infection, and wound dehiscence | the radiological evaluation was examined by CBCT and OPG and showed favorable bone healing after one year | NA |
